# Valonea Tannin: Tyrosinase Inhibition Activity, Structural Elucidation and Insights into the Inhibition Mechanism

**DOI:** 10.3390/molecules26092747

**Published:** 2021-05-07

**Authors:** Jiaman Liu, Yuqing Liu, Xiaofeng He, Bo Teng, Jacqui M. McRae

**Affiliations:** 1 College of Science, Shantou University, Shantou 515063, China; 19jmliu1@stu.edu.cn (J.L.); 15yqliu2@stu.edu.cn (Y.L.); 19xfhe@stu.edu.cn (X.H.); 2 Guangdong Provincial Key Laboratory of Marine Biotechnology, Shantou University, Shantou 515063, China; 3 School of Chemical Engineering and Advanced Materials, The University of Adelaide, Adelaide, SA 5005, Australia; jacqui.mcrae@adelaide.edu.au

**Keywords:** valonea tannin, hydrolysable tannin, tyrosinase, enzyme binding, enzyme inhibition, inhibition mechanism

## Abstract

Valonea tannin is a natural product readily extracted from acorn shells that has been suggested to have potential skin whitening properties. This study investigated the tyrosinase inhibition activity of extracted valonea tannin and the associated structure–function activity. Nuclear magnetic resonance spectroscopy and molecular weight analysis with gel permeation chromatography revealed that valonea tannin could be characterized as a hydrolysable tannin with galloyl, hexahydroxydiphenoyl and open formed-glucose moieties and an average molecular weight of 3042 ± 15 Da. Tyrosinase inhibition assays demonstrated that valonea tannin was 334 times more effective than gallic acid and 3.4 times more effective than tannic acid, which may relate to the larger molecular size. Kinetic studies of the inhibition reactions indicated that valonea tannin provided tyrosinase inhibition through mixed competitive–uncompetitive way. Stern–Volmer fitted fluorescence quenching analysis, isothermal titration calorimetry analysis and in silico molecule docking showed valonea tannin non-selectively bound to the surface of tyrosinase via hydrogen bonds and hydrophobic interactions. Inductively coupled plasma-optical emission spectroscopy and free radical scavenging assays indicated the valonea tannin had copper ion chelating and antioxidant ability, which may also contribute to inhibition activity. These results demonstrated the structure–function activity of valonea tannin as a highly effective natural tyrosinase inhibitor that may have commercial application in dermatological medicines or cosmetic products.

## 1. Introduction

Valonea tannin is a hydrolysable tannin [1] with many uses, including as wood adhesives [2], metal chelating depressants in the mining industry [3], leather tanning or potentially as a replacement for plastics [4]. Valonea tannin is abundantly present in the acorn cups of Valonia oak (*Quercus ithaburensis* subsp. *macrolepis* (Kotschy, Hedge and Yaltirik), a common tree throughout Eurasia, and can be readily extracted using hot water [5]. These properties make it a potentially useful resource for many applications that might provide greater economic value. 

Skin whitening agents are in large demand around the world with almost 15% of the human population investing in these products and the worldwide market reaching U.S. $23 billion in 2020 [6]. These agents are commonly used to treat a range of dermatological problems caused by the over-production of melanin pigment (known as disordered melanogenesis), such as melanoma, melasma, solar lentigo, freckles, pigmented acne scars and age spots [7,8]. Tyrosinase is a reaction rate-limiting enzyme of melanin synthesis and is also a target enzyme for the treatment of pigmentation related disorders [6,9,10]. Chemicals with tyrosinase inhibition abilities, such as hydroquinone, arbutin, kojic acid, corticosteroids, azelaic acid and hydroxyanisole, are widely applied in medicine and cosmetics as functional ingredients [6,9,10]. However, application of these chemicals is often associated with drawbacks and side effects, including contact dermatitis, irritation, burning and hypochromia [11,12]. Thereby, finding new tyrosinase inhibitors, with effective performance but without safety issues, has become a great concern for both the medical and cosmetic industries. 

**Figure 1:**
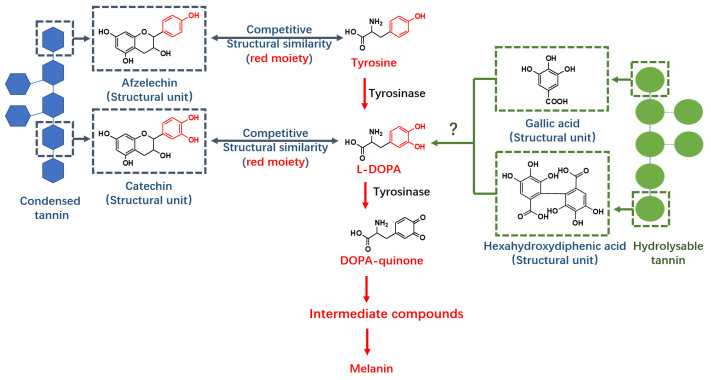
Condensed tannin has structural similarities with tyrosine and L-DOPA and thereby provides tyrosinase inhibition ability, but the structure of hydrolysable tannin differs from condensed tannin and its tyrosinase inhibition mechanism remains unknown.

Tannins are quantitatively abundant plant secondary metabolites that have protein binding capabilities. They are classified as either condensed or hydrolysable tannins depending on their molecular structure. Hydrolysable tannins are composed of glucose, galloyl, hexahydroxydiphenoyl (HHDP) and other derivatives subdivided into ellagitannin and gallotannin structures [1]. In contrast, condensed tannins (also known as proanthocyanidins) are composed of flavan-3-ol subunits which are linked by covalent bonds [13]. Condensed tannins have tyrosinase inhibition abilities which are comparable to commercial inhibitors [14,15]. This tyrosinase inhibition activity may be due to structural similarities between condensed tannin subunits and substrates (tyrosine and L-DOPA) (Figure 1) [15]. The mechanism of action may also be because condensed tannins behave as competitors in both L-DOPA and DOPA quinone formation processes [16]. 

Hydrolysable tannins may also have tyrosinase inhibition activity due to their protein binding capabilities. Previous studies have shown that the structural subunits of hydrolysable tannins, ellagic acid and gallic acid, have significant tyrosinase inhibition abilities [17]; however, the mechanism of action remains unknown. It is therefore likely that valonea tannin, a hydrolysable tannin, may exhibit tyrosinase inhibition activity, providing a novel, abundant and natural source of these important medical and cosmetic agents. 

In order to provide more theoretical information about the tyrosinase inhibition ability from hydrolysable tannin, also to extend the potential application of hydrolysable tannins. In the current study, the structures of valonea tannins extracted from acorn cups were elucidated and the tyrosinase inhibition activity compared to gallic acid and tannic acid. The inhibition mechanism was explored on the aspects of: (1) inhibition kinetic analysis; (2) tyrosinase binding ability; (3) antioxidant activity; and (4) copper ion (coenzyme) chelating ability, using fluorescence quenching accompanied with Stern–Volmer fitting analysis, isothermal titration calorimetry (ITC) analysis, as well as in silico molecular docking (Autodock Vina). 

## 2. Results and Discussion

### 2.1. Structure Elucidation of Valonea Tannins

Tannin is a mixture of oligomers and polymers with similar structures and physical properties [1]. Composition of structural moieties and molecular weights were considered as crucial structural characteristics because these characters have a substantial impact on the functions of tannins [18]. The valonea tannin was purified with Sephadex LH-20 because it was proven previously that this method can eliminate the simple phenolics, proteins and polysaccharides efficiently, therefore it commonly used for tannin purification [19]. Then, valonea tannin structure was elucidated using ^13^C NMR (Figure S1) to obtain the composition of the structural moieties. Chemical shifts were compared with those of standard tannic and gallic acids as well as those of similar moieties from previous reports [20] to give the proposed structure of valonea tannin based on the previous 1D and 2D-NMR research of ellagitannins and isolated flavonoid oligomers; resonances from 170 to 55 ppm were assigned to the carbons on carbonyl, phenyl and alkyl moieties, respectively, and shown on Table 1. 

Resonances from 100 to 170 ppm were used to identify the composition of hydrolysable tannins [20]. The chemical shifts appeared at 164.41, 158.50, 114.89 and 113.63 ppm were attributed to the carbonyl C=O and phenolic C-C bridges on hexahydroxydiphenoyl (HHDP) moieties [20]. These shifts can be considered as direct evidence to prove the existence of HHDP in valonea tannins. It also implied that valonea tannin belonged to the typical ellagitannin in the hydrolysable tannin family. This finding is in agreement with the report from Özgünay et al., who also found the HHDP moieties based on precursor and fragments ions in MALDI TOF MS spectrum of the valonea tannin [21].

Resonances at 163.98, 144.67 and 109.61 ppm were attributed to the carbonyl C=O, phenolic –OH and phenolic carbon on galloyl moieties [20]. Similarly, these shifts can be also found in gallic acid (169.1, 144.9 and 109.0 ppm) and tannic acid (169.7, 144.9 and 109.1 ppm). 

The resonances from 70 to 54 ppm, were characteristic resonances from the open glucose forms [22], while the closed glucose form (95 to 80 ppm) was not detected in the current study. Other resonances were attributed to the phenolic carbon linked with or without hydroxyl groups and were attributed to both HHDP and galloyl moieties [20]. 

**Figure 2:**
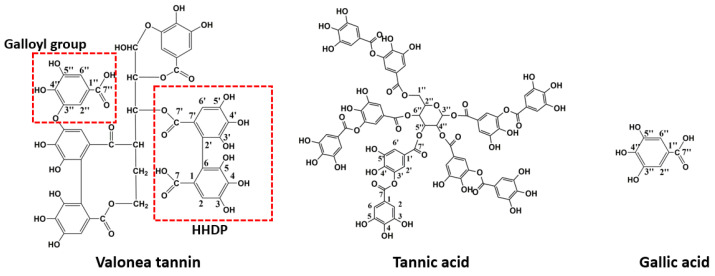
Example of the elucidated structure of valonea tannin, ^13^C NMR spectra indicate that tannic acid and gallic acid were dominant moieties in valonea tannin.

These chemical shifts not only confirmed the existence of galloyl and HHDP moieties in valonea tannins, but also indicated the structural characteristics of the valonea tannins were significantly different to condensed tannins (composed of flavanol subunits) [23]. Based on the information obtained from ^13^C NMR analysis, the typical structure of the valonea tannin was deduced and shown on Figure 2, accompanied with the structure of tannic acid and gallic acid. 

**Figure 3:**
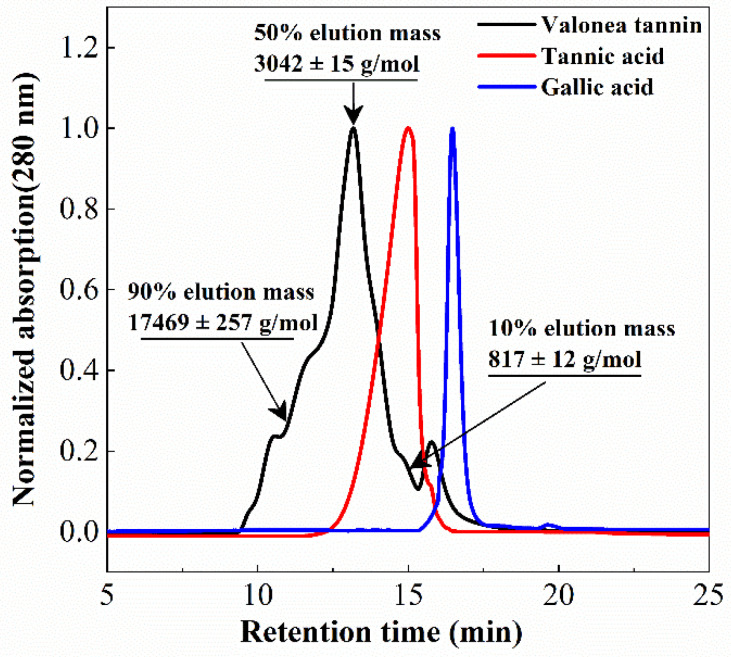
Gel permeation chromatogram (280 nm) showed the elution profile of valonea tannin, tannic acid and gallic acid, average molecular mass of valonea tannin is shown as the 50% elution mass.

Tannin molecular weight is directly related to the biochemical properties and is a crucial parameter for protein interaction and enzyme inhibition [15]. Determining the average molecular weight of a tannin can also assist in elucidating the average size of the tannins [15,18]. The molecular weight distribution of the isolated valonea tannin was analysed using gel permeation chromatography (GPC) (Figure 3). 

Based on the GPC results, 10%, 50% and 90% elution mass of the valonea tannins were 817 ± 12, 3042 ± 15 and 17,469 ± 257 g/mol, respectively. This indicated that 80% of the tannin molecules had a mass ranging from 817 to 17,469 g/mol. Furthermore, 50% valonea tannin molecules eluted earlier than tannic acid and 90% of the valonea tannin showed higher molecular weight than gallic acid. This indicated the average molecular weight of valonea tannin should be higher than the other two compounds. Based on the results from Kennedy’s report, 50% elution mass of tannin is in good agreement with the average molecular weight determined by other method [19]. Therefore, the 50% elution mass (3042 ± 15 g/mol) of the valonea tannin was taken for the following analysis. 

### 2.2. Tyrosinase Inhibition Activity of the Valonea Tannin

Valonea tannin was assessed for tyrosinase inhibition to determine if this natural product may have value for commercial application as a whitening agent in cosmetic products or dermatologic medicines. Tannic acid and gallic acid were also assessed for tyrosinase inhibition for comparison as these compounds were: (1) all composed of pyrogallol moieties (Figure 2) that played critical roles in the enzyme inhibition processes [9]; (2) have molecular weights that differ to each other and are lower than valonea tannins (Figure 3); and (3) accepted as tyrosinase inhibitors with better performances than the commercial tyrosinase inhibitors [9]. 

The tyrosinase inhibition activities were assessed across a range of concentrations for valonea tannins as well as tannic and gallic acids. Inhibition was measured as an increase in absorbance over time (Figure S2). The IC_50_ values were calculated as the inhibitor concentration inducing a 50% reduction in maximum absorbance and are presented in Table 2. 

The IC_50_ values of the involved inhibitors followed a trend: valonea tannin < tannic acid < gallic acid. This suggested that tyrosinase inhibition capability was, positively related to molecular weight, which is also in agreement with a conclusion obtained by condensed tannins with different molecular weights [15]. 

Gallic acid has previously been shown to have excellent tyrosinase inhibition ability with an IC_50_ value 100-fold lower than kojic acid [24]. In the current study, the IC_50_ value of valonea tannin was 339-fold lower than gallic acid (Table 2), which indicated that the valonea tannin may has better tyrosinase inhibition ability than the commercial inhibitors. To explore why the valonea tannin showed such an outstanding tyrosinase inhibition ability, the inhibition mechanisms were studied and presented in the following sections.

### 2.3. The Kinetic Study of Tyrosinase Inhibition Mechanism

**Figure 4:**
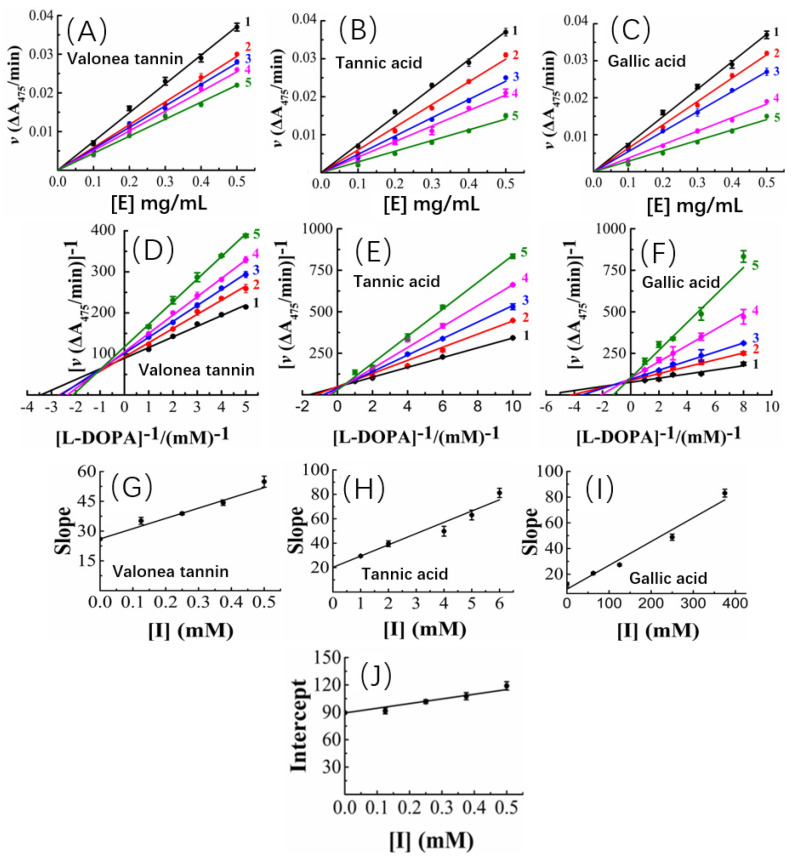
Tyrosinase concentration-reaction rate plots showed the valonea tannin (**A**), tannic acid (**B**) and gallic acid (**C**) reversibly inhibited tyrosinase activities (1 to 5, gradually increase the concentration of inhibitors); The Lineweaver–Burk plots of valonea tannin (**D**), tannic acid (**E**) and gallic acid (**F**); the plot of slope (or intercept) versus inhibitor concentrations of valonea tannin (**G**), tannic acid (**H**) and gallic acid (**I**); the plot of intercept versus valonea tannin concentration for determining inhibition constants K_IS_ (**J**).

To answer why valonea tannin has inhibition ability, kinetic characteristics of the inhibition reactions were studied to better understand the mechanism of action of valonea tannin’s tyrosinase inhibition. The kinetic studies were applied and the results are provided in Figure 4.

The tyrosinase concentration-reaction rate plots showed that, with a constant valonea tannin concentration, the initial velocities linearly fitted with tyrosinase concentrations and the fitted lines all passed through origin of the axis (Figure 4A). Therefore, a deduction can be made as: despite varying valonea tannin concentrations, the reactions will continue proceeding until tyrosinase is eliminated completely from the reaction systems ([E] = 0). Based on the characteristics mentioned above, referencing the previous reports [16,25], the valonea tannin inhibition reaction was classified as a reversible type. As for tannic acid or gallic acid, their tyrosinase concentration-reaction rate plots also showed significant characteristics of the reversible type as well (Figure 4B,C), in agreement with the results obtained by previous research [26]. 

Based on the adjusted Michaelis–Menten equation [27], the Lineweaver–Burk plots of the reactions were obtained by analysing velocity under different valonea tannin concentrations (Figure 4D). It was evident that the inverse of the velocity (1/v) were all linearly fitted with the inverse of the substrate concentration (R^2^ and *p*-values are shown in Table S4) while meanwhile it intersected in the second quadrant of the plot. Based on Waldrop’s finding, these graphical characters indicated the maximum reaction speed (Vmax = 1/intersect on y-axis) and Michaelis’s constant (K_M_) of the reactions were all affected with varying inhibitor concentrations [27]. Thus, the inhibition provided by valonea tannins belonged to a competitive−noncompetitive mixed type. In other words, the valonea tannin presents inhibition through either entering the active centre or binding on the surface (non-active centre) of tyrosinase. This is similar to the inhibition mechanism provided by condensed tannins [16]. In contrast to valonea tannins, the Lineweaver–Burk plots of the tannic acid and gallic acid showed different characteristics (Figure 4E,F). The fitted lines were intersected on y-axis, which indicated these compounds inhibit tyrosinase competitively, which is in agreement with a previous report [28].

Slopes and y-axis intercepts of the fitted Lineweaver–Burk lines were taken for secondary fittings, where they all were linearly fitted with inhibitor concentrations (Figure 4G–I). The slopes and y-axis intercepts of the new lines were calculated to obtain the inhibition constants of inhibitor-tyrosinase (K_I_) and inhibitor–tyrosinase–substrate complexes (K_IS_) (Figure 4J), respectively [27]. K_I_ is the dissociation constant between inhibitor and enzyme, and K_IS_ is the dissociation constant between inhibitor and the enzyme–substrate complex. The smaller the K_I_ or K_IS_ values provided by inhibitor, the better competition abilities to substrate were indicated [27]. 

In the current research, the K_I_ and K_IS_ values of the involved inhibitors followed a trend as: valonea tannin (K_I_) < valonea tannin (K_IS_) < tannic acid (K_I_) < gallic acid (K_I_) (Table 3). Indicated in comparison with tannic acid and gallic acid, the valonea tannin showed better competition ability than substrate. Furthermore, the higher the molecular weight of tested inhibitors, the better the competition ability that was shown. This trend is not only in agreement with the results observed from IC_50_, but also similar to the conclusions that were obtained from condensed tannins [29]. The results obtained from the kinetic study are summarized on Table 3.

To find more information to explain why valonea tannin showed a better competition ability to substrate, the mechanism of valonea tannin–tyrosinase interactions were investigated using fluorescence and isothermal titration calorimetry (ITC) as described below.

### 2.4. Fluorescence and Thermodynamic Characters of the Valonea Tannin-Tyrosinase Interaction

**Figure 5:**
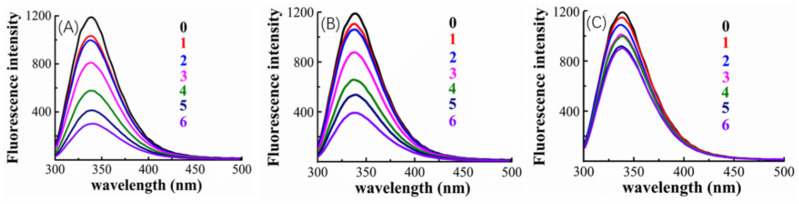
Fluorescence emission spectra of mushroom tyrosinase in solutions combined with valonea tannin (**A**), tannic acid (**B**) and gallic acid (**C**) with a range of concentrations (0 = 0 μM control; 1–6 = 5, 10, 25, 50, 75 and 100 μM, respectively).

**Figure 6:**
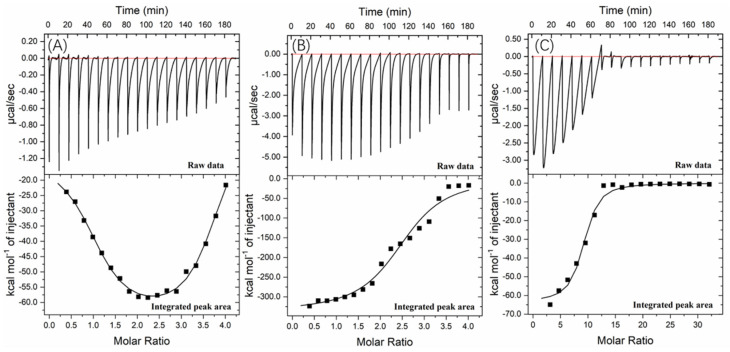
Interaction of (**A**) valonea tannin, (**B**) tannic acid and (**C**) gallic acid with tyrosinase studied by ITC at 25 °C showed the thermogram and binding isotherm, the titration curve from valonea tannin was fitted using two sets of sites binding model while tannic acid and gallic acid were fitted by one set of sites.

The cause of the observed improved competition ability of valonea tannin was explored using fluorescence quenching (Figure 5) and isothermal titration calorimetry (ITC) (Figure 6). 

The tryptophan inside tyrosinase is the fluorophore that has florescence emitting with a 280 nm excitation wavelength [30]. The tyrosinase–inhibitor binding process may lead to a fluorescence quenching induced by the structural changes of the tyrosinase around tryptophan, consequently, fluorescence quenching was used to provide primary information about inhibitor–tyrosinase interaction [30].

Figure 5 indicated that, the tyrosinase showed a florescence emission at 340 nm, and fluorescence quenching appeared after adding inhibitors (valonea tannin, tannic acid or gallic acid, respectively). Greater concentrations of inhibitors also increased the observed quenching, which implies that the quenching may be induced by the interaction between inhibitors and tyrosinase. 

The quenching phenomenon can be classified into dynamic and static types, while the former one does not involve chemical interaction between fluorophore and quencher, only the static quenching refers to binding interactions [29]. Based on the study provided by Van De Weert and Stella [29], the Stern–Volmer quenching rate constant (K_q_) and apparent binding constant (K_a_) were consequently obtained by linear regressions of the Stem–Volmer equation and double log Stem–Volmer equation (Figure S3) and shown in Table 4.

The fluorescence analysis results showed that K_q_ presented by valonea tannin–tyrosinase complex was significantly higher than the maximum scatter collision quenching rate constant for dynamic quenching (2 × 10^10^ L/mol/s) [29], meanwhile their maximum emission wavelengths were observed as constant at 340 nm (Table 4). This phenomenon implied a non-covalent bond was formed between valonea tannin and tyrosinase [29], which is in agreement with the results obtained through hydrolysable tannin–protein interactions [31]. As for the tannic acid and gallic acid, the tyrosinase showed similar quenching characters. This further suggests that these interactions were non-covalent as previously reported for interactions between tyrosinase and tannic acid [32]. The quenching efficiency as well as the K_a_ of the involved inhibitors followed: valonea tannin > tannic acid > gallic acid. These results preliminary indicated the valonea tannin maybe more strongly bound to tyrosinase than the other two inhibitors [29].

Further information about the valonea tannin–tyrosinase binding process was further investigated using ITC analysis. The tyrosinase is a typical globular protein which has complicated stereochemical structures, and tannin is a natural product with polydispersity, therefore tannin–protein interactions are believed to be complex [33]. ITC enables measurement of the binding strength of tannin–protein interactions by quantifying the thermodynamic changes of complex reactions [34]. The ITC curves of valonea tannin, tannic acid and gallic acid titrated into tyrosinase are shown in Figure 6. These negative peaks, shown on the raw data, were attributed to the negative enthalpy from tannin–tyrosinase interaction. In other words, the interaction was exothermic [33]. These titration signals were typical of protein–ligand interactions, resulting from the combination of hydrogen bonding and hydrophobic interactions as has been previously seen in the initial section of ITC curves from earlier reports [34]. 

The rapid reduction of exothermic peaks (ΔH < 0) upon titration indicated the valonea tannins showed a strong affinity to tyrosinase. With increasing valonea tannin addition, the number of available binding sites decreased, which resulted in the associated enthalpy changes decreasing. The titration curve was found to better fitted with the “two sets of binding sites” model, which implied the two kinds of binding sites were occupied by valonea tannins. On the contrary, the titration curves obtained from tannic acid and gallic acid showed better fitted with the “one set of binding sites” model. The inhibition mechanism results classified valonea tannin as a competitive–noncompetitive mixed type, while classified gallic acid and tannic acid were competitive types (Table 3). This also indicated the valonea tannin bound on both active and non-active sites of the tyrosinase, while gallic acid and tannic acid bound on the active sites further confirming the proposed inhibition mechanism.

Enthalpy change (ΔH) was calculated from the area under each ITC curve, and entropy changes (TΔS), stoichiometry (molar ratio of inhibitor to site, expressed as “n” [34]) and equilibrium binding constants (K) were calculated from the graphical characters of the curve using the Origin 7.0 software package. Results are shown in Table 5. The negative ΔH values of valonea tannin–tyrosinase interaction indicating that interactions on these two sites were exothermic. The negative ΔH and TΔS, observed from inhibitor–tyrosinase complexes, demonstrate both hydrophobic interactions and hydrogen bonding were essential for the binding [34].

The valonea tannin showed K values 70.6 ± 3.0 (×10^5^) at binding site 1 and 4.3 ± 0.8 (×10^5^) at binding site 2, which were significantly higher than the tannic or gallic acids, suggesting stronger binding. This is likely to relate to the greater molecular weight of the valonea tannin that enabled quicker and stronger inhibition to tyrosinase. As expected, the n values of valonea tannins were smaller than (or similar to) tannic acid and gallic acid. This phenomenon could be attributed to the stronger steric hinderance, larger hydrodynamic volumes and higher binding strength presented by valonea tannin, which were obtained from GPC, inhibition kinetic and fluorescence study. This result supports the trend observed in apparent binding constant (K_a_, from Stern–Volmer quenching) and inhibition constant in enzyme−substrate complex (K_IS,_ from Michaelis–Menten kinetic) in this study, as well as a previous hypotheses that considers molecular weight as a key influence of tannin–protein interaction [35].

### 2.5. Molecular Docking Analysis

**Figure 7:**
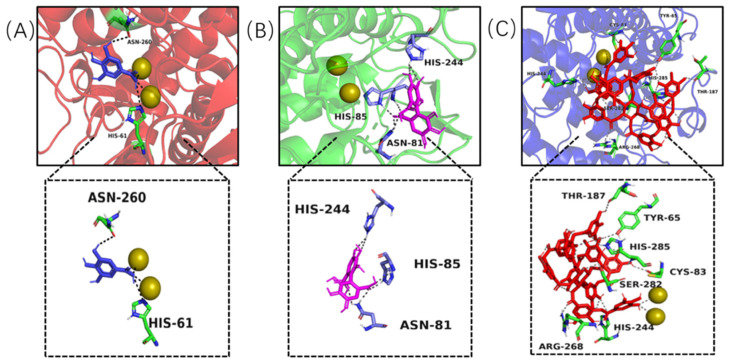
Complexes formed by tyrosinase and ligands and observed by molecular docking, including gallic acid–tyrosinase (**A**), HHDP–tyrosinase (**B**) and tannic acid–tyrosinase (**C**), the yellow and spheroidal icons were copper ions inside active central of the tyrosinase, the dotted lines were hydrogen bond formed between ligand and tyrosinase.

Molecular docking modelling was performed to provide insight to the specific tyrosinase inhibition activity of valonea tannin. As revealed by ^13^C NMR, the valonea tannins were composed of galloyl and HHDP as structural units. Thereby the gallic acid and HHDP were chosen as ligand models and applied for molecular docking analysis. For the docking results from each ligand-tyrosinase interaction, 20 complexes with different docking poses (also with lowest docking energies) were collected, among which the complexes with lowest energies are shown on Figure 7.

Molecular docking results indicated the model molecules (including gallic acid and HHDP) bind with tyrosinase at the active centre via hydrogen bonding [36]. Two hydrogen bonds were observed (Asn260 and His61) in the gallic acid–tyrosinase complex. As for HHDP, six hydrogen bonds were formed via His85, Asn81 and His244 inside the active centre. As for the tannic acid–tyrosinase complex, more hydrogen bonds were observed (Figure 7C). Thr187, His285, Cys83, His244, Ser282, Tyr65 and Arg268 were shown participating in the interaction; meanwhile eight hydrogen bonds were formed. 

The results from molecular docking not only visualized the active site bindings which were observed from ITC and fluorescence analyses, but also discovered the interaction between copper ions and gallic or tannic acid. The valence states of these copper ions not only determine the type of activity showed by tyrosinase (diphenol or monophenol) but also believed to influence the activity of tyrosinase in catalytic cycles [37]. Therefore, the copper ion chelating and antioxidant ability of the valonea tannin were assessed and presented in the following section.

### 2.6. Copper Ion Chelating and Antioxidant Abilities

To further explore the inhibition mechanism proposed by the molecular docking studies, copper chelation and antioxidant analyses were also conducted (Table 6).

The adjacent hydroxyl groups on galloyl moieties, enabled the hydrolysable tannin to chelate with metal ions and form aggregates and eventually precipitate [35]. Copper ion chelating ability was analysed using inductively coupled plasma (ICP) spectroscopy and shown in Table 6. The results indicated that, after reacting with valonea tannin, large quantities of copper ions were precipitated and only 56.61 ± 0.66% were remaining in the aqueous phase. This implied chelating with copper ion was another pathway of tyrosinase inhibition, in agreement with the phenomenon observed through molecular docking. 

Chemicals with antioxidant activities are known to also have tyrosinase inhibition activities, not only because the tyrosinase catalysed L-DOPA to DOPA quinone reaction is basically an oxidation reaction, but also because the antioxidants may have an impact on the oxidation state of copper ions thereby becoming a hindrance in catalytic cycles of tyrosinase [37]. The DPPH^·^ and ABTS^·+^ scavenging abilities of valonea were compared to that of a known antioxidant, ascorbic acid (Table 6). Valonea tannin provided significantly lower IC_50_ values than ascorbic acid, which indicated substantial antioxidant capability that may also contribute to the tyrosinase inhibition ability, as previously noted for ascorbic acid [38].

In the current study, the tyrosinase inhibition activity of the inhibitors followed: valonea tannin > tannic acid > gallic acid. This trend repeatedly appeared in the results observed from tyrosinase binding analysis, as for the antioxidant and copper chelating abilities, the valonea tannin still showed better performance than the gallic acid and ascorbic acid but was similar to tannic acid (Table 6). Therefore, the outstanding tyrosinase inhibition provided by valonea tannins is basically attribute to its tyrosinase binding ability.

## 3. Materials and Methods

### 3.1. Extraction and Purification of Valonea Tannins

Acorn caps (valonea, 600 g) were collected from Valonia Oak trees (*Quercus macrolepis*) in Wuming (108°16′27.34″ E, 23°8′36.66″ N, South China) in 2019. Valonea were washed with distilled water, air-dried at 25 °C and ground into a fine powder using a plant tissue pulveriser (800A, Jinfeng Machinery Factory, Yongkang, China). Tannins were extracted from the powder (100 g) using 70% acetone: water solution (2.0 L) in a temperature-controlled shake incubator (THZ-C-1, Taicang experimental equipment factory, Hebei, China) with rotation speed at 30 rpm, 25 °C for 8 h. After extraction, the valonea residue was removed via filter paper filtration, and acetone removed by rotary evaporation under reduced pressure (35 °C) to obtain a crude aqueous extract (140 mL). Lipids were removed from the aqueous extract by liquid–liquid separation via the addition of dichloromethane (1:1, *v*:*v*). Finally, the residual organic solvent was removed from the aqueous extract by rotary evaporation (35 °C) and the extract lyophilized to obtain an 80 g crude extract of powdered valonea tannins.

Crude valonea extracts (1 g in 10 mL 50% MeOH solution) was purified with Sephadex LH-20 (Cytiva, Switzerland), as previously described [18]. Briefly, the Sephadex LH-20 column (φ = 3.5 cm, bed volume 260 mL) was equilibrated with 50% MeOH (1% *v*/*v* formic acid) prior to loading the extract. Sugars and simple phenolics were removed using 50% MeOH solution (5 L, flow rate 10 mL/min) and purified tannin eluted with 70% acetone (1.5 L), dried via rotary evaporation (35 °C) followed by lyophilization to obtain the purified valonea tannin powder (240 mg). Purity of the tannin extract was confirmed using MALDI-TOF MS analysis, which demonstrated the absence of polysaccharides, proteins and lipids (Figure S5). The tannin purity was also tested by a methyl cellulose precipitation assay [39] and the results showed tannin concentration was 81.85 ± 0.93% (standard curve is shown in Figure S6).

### 3.2. Characterization of Valonea Tannin Structure

Characterization of the purified tannin structure was achieved using Nuclear Magnetic Resonance (NMR) spectroscopy and the average molecular mass measured using gel permeation chromatography (GPC). For the NMR analysis, purified valonea tannin powder (30 mg in 750 μL 1:1, *v*/*v*, CD_4_O:D_2_O) ^13^C NMR spectra were obtained using an Ascend 400 MHz NMR spectrometer (Bruker, Switzerland) [20], 1.36 s acquisition time, 20.80 μs dwell time, sweep width 24,038 Hz, frequency 100.60 MHz, relaxation delay 2 s, receiver gain 203. Power level for pulse was set as 66 W and 90° high power pulse was set at 10.57 μs. The ^13^C NMR spectra were also obtained for standard samples of gallic acid (Aladdin Biochemical Technology Co., LTD, Shanghai, China) and tannic acid (Macklin Biochemical Technology Co., LTD, Shanghai, China), and chemical shifts were assigned (Figure S1). GPC was used to obtain the average molecular mass of the purified tannin as previously described [18,19]. Briefly, the tannin sample (20 μL in mobile phase) was injected into 2 x PLgel GPC columns in tandem (300 × 7.5 mm, 5 μm, 100 Å followed by 10^4^ Å) on an HPLC (Agilent 1100, Palo Alto, USA) and protected by a guard column of the same material (50 × 7.5 mm, 5 μm) using a mobile phase of dimethylformamide solution (1% acetic acid, 5% water and 0.15 M lithium chloride) at 1 mL/min, column temperature 50 °C, with detection at 280 nm. The average molecular mass of valonea tannin was obtained by comparing elution time with a standard curve made by isolated grape skin tannins with molecular weight 2035 g/mol, 5016 g/mol, 7993 g/mol and 17,674 g/mol, respectively. These grape tannins were prepared in accordance with our previous report [18]. The molecular weights of the grape tannins were determined with phloroglucinolysis in accordance with Kennedy’s report [19].

### 3.3. Tyrosinase Inhibition Activity Assessment and Inhibition Type Analysis

Tyrosinase inhibition activity was evaluated as previously described [17] with L-DOPA as the substrate and the enzyme activity calculated by monitoring DOPA-quinone formation. Tyrosinase (EC 1.14.18.1, from mushroom) and L-DOPA (both from Aladdin Biochemical Technology Co., LTD, Shanghai, China) were dissolved in the sodium phosphate buffer (PBS, 50 mM sodium phosphate in distil water, pH = 6.8, same as below), respectively, to obtain L-DOPA solution (0.5 mM) and tyrosinase solution (0.4 mg/mL). Valonea tannin was prepared in water to a range of concentrations (0 mM, 0.2 mM, 0.4 mM, 0.6 mM, 0.8 mM, 1.0 mM, 1.3 mM and 1.5 mM). For the inhibition assays, valonea tannin solutions (50 μL) at each concentration were mixed with L-DOPA solution (1 mL) and heated in a water bath (30 °C) for 10 min. Tyrosinase solution (50 μL) was then added to each solution, mixed and an aliquot (300 μL) immediately transferred into a 96-well plate. Absorbances were measured at 475 nm at the beginning (0 min) and end (10 min) of the reactions using a microplate reader (HTX, Bior Tek Synergy, Vermont, USA). During the analysis, the temperature was kept constant at 30 °C by using a temperature controller equipped on the microplate reader. Reaction solution without tannin was used as negative control. The inhibition rate was calculated in accordance with Equation (1).

Inhibition rate (%) = [(A_2_ − A_1_) − (B_2_ − B_1_)]/(A_2_ − A_1_) × 100% (1)

where A_1_ and A_2_ represented the absorbance of the control at the beginning and end of the reaction; B_1_ and B_2_ represented the absorbance of the sample at beginning and end of the reaction, respectively.

The extent of inhibition was calculated using the obtained inhibition rate-inhibitor concentration plots (Figure S2) and expressed as the half maximal inhibitory concentration (IC_50_).

Inhibition type was analysed based on the kinetic characteristics of the DOPA-quinone formation processes, which is concomitant with valonea tannins. The reversible–irreversible assessment was provided as previously described [16] with modifications. The initial velocities of DOPA-quinone formations were tested in reaction solutions with a constant L-DOPA concentration (0.5 mM), and different valonea tannin (0 mM, 0.125 mM, 0.25 mM, 0.375 mM and 0.5 mM) and tyrosinase concentrations (0.1 mg/mL, 0.2 mg/mL, 0.3 mg/mL, 0.4 mg/mL and 0.5 mg/mL). The absorbances of reaction solutions were recorded at 475 nm and the initial velocities were taken and expressed as ΔA/min.

The competitive–noncompetitive assessment was performed in accordance with a previous report [25]. Briefly, initial velocities (recorded at 475 nm) of reactions were taken from solutions prepared using a constant tyrosinase concentration (0.1 mg/mL), and different valonea tannin (0 mM, 0.125 mM, 0.25 mM, 0.375 mM and 0.5 mM) and L-DOPA concentrations (1 mM, 0.5 mM, 0.33 mM, 0.25 mM and 0.20 mM). The Michaelis–Menten equation was then adjusted to fit Lineweaver–Burk plot as Equation (2) [27]:

(1/V) = (K_M_/V_max_) × (1/[S]) + (1/V_max_) (2)

where [S] was the substrate concentration, V was the corresponding initial velocity. K_M_ and V_max_ were the Michaelis’s constant and maximum reaction speed, respectively, which were obtained from the vertical and horizontal intercept from the Lineweaver–Burk plot. 

The inhibition constant (K_I_) and inhibition constant in enzyme−substrate complex (K_IS_) were obtained by secondary plot of inhibitor concentration with slope and intercept of the Lineweaver–Burk plot as Equations (3) and (4) [27]:

Slope = K_M_/V_max_ (1 + [I]/K_I_) (3)

Intercept = 1/V_max_ (1 + [I]/K_IS_) (4)

The tyrosinase inhibition activity of tannic acid and gallic acid were also measured along with the inhibition type analysis for comparison with valonea tannin.

### 3.4. Fluorescence Quenching Analysis of Tyrosinase in the Presence of Valonea Tannins

Fluorescence quenching was used to measure the change in tyrosinase activity in the presence of valonea tannins. The analysis was conducted in accordance with a published protocol [30], and the method was adjusted as follows: The fluorescence intensities were recorded by a fluorescence spectrometer (Hitachi F-7000, Kyoto, Japan) equipped with a xenon lamp source. A 280 nm excitation wavelength, 240 nm/min scan speed, 5 nm emission slit width and 2.5 nm excitation slit width were chosen for the analysis. In this assay, valonea tannins with different concentrations (0, 5, 10, 25, 50, 75 and 100 μM) were mixed with tyrosinase solution at a fixed concentration (0.4 mg/mL). The prepared samples were then transferred for fluorescence analysis, while intensities were recorded from 300 to 500 nm. In the current study, emissions at 340 nm were chosen for quenching related calculations since the maximum fluorescence appeared at this wavelength. Quenching was calculated using the Stern–Volmer [29] Equation (5):

F_0_/F = 1 + K_sv_ [Q] = 1 + K_q_τ_0_ [Q] (5)

where F and F_0_ were the fluorescence intensities (at 340 nm) with or without valonea tannin. [Q] was the tannin concentration and K_sv_ is the Stern–Volmer quenching constant. The K_sv_ was the slope calculated from the linear regression plot of F_0_/F against [Q]. K_q_ was the biomolecular quenching rate constant. τ_0_ was the average lifetime of the fluorophore in the absence of the tannin (τ_0_ = 10^−8^ s) [29]. 

As for the static quenching, the F_0_, F and [Q] were taken for apparent binding constant calculation based on double log Stern–Volmer [29] Equation (6):

Log [(F_0_ − F)/F] = log K_a_ + nlog [Q] (6)

where K_a_ was the apparent binding constant that was obtained by the slope of log [(F_0_ − F)/F] versus log [Q] plots. Double log plotting needed restrict requisite otherwise false n values could be obtained [29]. Therefore, in the current study, the n value was not taken into consideration. The fluorescence quenching of tyrosinase was analysed with gallic acid and tannic acid for comparison, the Stern–Volmer plot (F_0_/F against [Q]) and double log Stern–Volmer plot (log [(F_0_ − F)/F] versus log [Q]) are provided in Supplementary Information S3.

### 3.5. Isothermal Titration Calorimetry Analysis

Isothermal Titration Calorimetry (ITC) analysis was conducted as previously described [34] but modified as follows: Tyrosinase solution (50 μM PBS buffer 50 mM, pH = 6.8) added to the calorimeter cell (200 μL) on an ITC instrument (ITC 200, MicroCal, Northampton, MA, USA) instrument and equilibrated at 25 °C for 30 min under a rotation speed of 1000 r/min. To calculate the thermal character during binding, valonea tannin solution (1 mM in PBS buffer 50 mM, pH = 6.8) was injected into the sample cell at 25 °C. The injection volume, number of injections and the spacing time between injections were set as 2 μL, 18 and 600 s, respectively. During analysis, the valonea tannin solution was injected into the reference cell which contained a buffer solution; the thermal behaviour was recorded and used as a negative control.

Peak interpretation, stoichiometry (n), the binding constant (K), change in enthalpy (ΔH) and change in entropy (ΔS) were calculated on Origin 7.0 software package (Northampton, MA, USA), while changes in Gibbs free energy were calculated based on a previously report [40]. A “two set of identical sites” model was applied for valonea tannins. The tannic acid–tyrosinase reaction and gallic acid-tyrosinase reaction were also analysed as described above, and the data were fitted with the “one set of identical sites”. All standard deviations shown in the ITC results were based on the accuracy of the curve fit to the data and obtained by Origin software package. 

### 3.6. In Silico Molecule Docking

In silico molecule docking models were performed using AutoDock Vinasoftware (DeLano Scientific LLC, Palo Alto, CA, USA) to provide an understanding of the mechanism of tyrosinase inhibition by valonea tannins. The crystallographic structure of the tyrosinase–tropolone complex from *Agaricus bisporus* (PDB: 2Y9X, obtained by X-ray diffusion) was obtained from RCSB Protein Databank. Then, tropolone and water molecules on tyrosinase were removed, followed by a polar hydrogen atoms addition, a missing atoms correction and a Gasteiger charges assignment [41]. Gallic acid, HHDP and tannic acid were constructed with Chem Draw 17.0 (Cambridge, UK) and taken as ligands. Structures of the ligands were subsequentially geometry optimized with an MM2 force field to minimize the energy and obtain the preferential conformations [41].

In accordance with Heitz’s report [41], the blind docking simulations were performed within a grid box which was centred on the geometric central position of the tropolone on tyrosinase complex (x, y, z: −7.392, −24.898, −39.626). The grid box was set to have a 30 × 30 × 30 size with grid spacing of 0.1 nm. The energy range was set at 10, while exhaustiveness was 20. Other operator weights for crossover, mutation and elitism were default parameters. A semi-flexible docking was chosen for all processes, while the sophisticated gradient optimization method in its local optimization was employed to search for the preferential conformations, and the empirical scoring function was used for docking score calculation. The predicted binding energies were collected from 20 ligand–receptor complexes with lowest energies, from which the complex with the lowest binding energy was preserved and pose of the ligand, hydrogen bond and related amino acid residuals were analysed with PyMOL 2.2 (Schrödinger Inc, New York, NY, USA). 

### 3.7. Antioxidant Activity Analysis

The antioxidant activity of the valonea tannin was evaluated using scavenging assays with 3-ethylbenzthiazolin-6-sulfonic acid (ABTS^·+^) as well as 2,2-diphenyl-1-picrylhydrazyl (DPPH^·^). The DPPH^·^ scavenging ability was analysed in accordance with Brand-Williams’s report [42]. DPPH^·^ solutions (1.5 mL, 25 mg/Lin methanol) were mixed with 50µL valonea tannin solutions at different concentrations (0 mM, 0.02 mM, 0.03 mM, 0.04 mM, 0.05 mM and 0.06 mM in methanol), and placed in the dark for 30 min. After the reaction time, 300 µL of the mixed solution was transferred into a 96 well microplate reader, and the absorbance measured at 517 nm and corrected using methanol. This method was used in place of the valonea tannins as a negative control.

The ABTS^·+^ scavenging ability was measured as previously described [43]. Briefly, 7 mM ABTS and 2.45 mM potassium persulfate were mixed and placed in the dark for 16 h (at 25 °C) to form a stable oxidation state of ABTS^·+^ radical cations. ABTS^·+^ solution was then diluted with 80% ethanol to reach an absorbance of 0.700 ± 0.05 at wavelength 734 nm. Valonea tannin solutions (0.1 mL) with different concentrations (0 mM, 0.02 mM, 0.03 mM, 0.04 mM, 0.05 mM, 0.06 mM and 0.07 mM, in 80% ethanol) were mixed with 3.9 mL ABTS^·+^ solution. After 6 min reaction (at 25 °C), 300 µL of the mixed solution was transferred into a 96 well microplate and the absorbance at 734 nm was recorded, corrected with methanol. Methanol (0.1 mL) was used to replace the valonea tannins as a negative control.

The DPPH^·^ and ABTS^·+^ free radical scavenging rates were calculated by using Equation (7):

Free radical elimination rate (%) = [(A_1_ − A_2_)/A_1_] × 100 (7)

where A_1_ was the absorbance of control, and A_2_ was the absorbance of sample.

The IC_50_ was obtained using the linear regression of a valonea tannin concentration to free radical elimination rate plot. Following the method described above, IC_50_ values were also obtained for gallic acid, tannic acid and ascorbic acid for comparison.

### 3.8. Inductively Coupled Plasma-Optical Emission Spectroscopy (ICP-OES)

The Cu^2+^-binding capacity of valonea tannin was measured using ICP-OES as previously described [44]. Valonea tannin solution (1 mg/mL, in distilled water) and copper sulphate solution (0.5 M, in distilled water) were mixed (1:1, v:v), incubated for 3 h (37 °C) and centrifuged for 30 min (4390 g). The supernatant was subjected to an ICP-OES analysis (Agilent 5110, Palo Alto, CA, USA) and the remnant Cu^2+^ concentration measured. A 12.0 L/min plasma flow rate, 1.5 L/min auxiliary flow rate and 0.70 L/min nebulizer flow rates were provided during the analysis, while sample uptake and instrument stabilization delay were 15 s. Gallic acid and tannic acid were also used to react with copper sulphate and remnant Cu^2+^ were quantified following above method for comparison.

### 3.9. Statistical Analysis

Non-parametric test (Kruskal–Wallis) was performed to determine differences between the experimental samples (triplicated) using Minitab 18 (Minitab Inc., State College, PA, USA). The model fitting results are all applied in Supplementary Information S4.

## 4. Conclusions

Elucidation of the isolated valonea tannin revealed the tannin was composed of gallic acid, HHDP and glucose with an average molecular weight of 3042 ± 15 Da. Tyrosinase inhibition ability provided by valonea tannin was 334 times greater than that of gallic acid and 3.4 times higher than tannic acid. Mechanism studies suggested that the observed enzyme inhibition was driven by a combination of hydrogen bonding and hydrophobic interactions which involved non-selective binding to the surface of tyrosinase, resulting in a competitive and non-competitive mixed inhibition mechanism. Antioxidant activity and copper ion chelating ability of valonea tannin also contributed to tyrosinase inhibition. These results suggest that extracted valonea tannins may be of value as a whitening agent in cosmetics or dermatological medicines as an effective replacement for commercial tyrosinase inhibitors.

## Figures and Tables

**Table 1:** ^13^C NMR chemical shifts assignment for the Valonea tannin ^1^.

Chemical Shift (ppm)	Assignment	Note
**Valonea tannin**		
164.41	C7″	Carbonyl C=O, HHDP
163.98	C7	Carbonyl C=O, galloyl
158.50	C7′	Carbonyl C=O, HHDP
144.67	C3	Phenolic –OH, galloyl
143.65	C5, C3′, C5′, C3″, C5″	Phenolic –OH, galloyl and HHDP
135.92	C4, C4′, C4″	Phenolic –OH, galloyl and HHDP
123.88	C1, C1′, C1″	Phenolic carbons, galloyl and HHDP
114.89	C2′	Phenolic C-C bridges, HHDP
113.63	C2″	Phenolic C-C bridges, HHDP
109.61	C2, C6	Phenolic carbons, Galloyl
70.04	C-5‴	Open glucose form
62.71	C-3‴, C-4‴, C-6‴	Open glucose form
57.40	C-2‴	Open glucose form
**Gallic acid**		
169.1	C7	Carbonyl C=O
144.9	C3, C5	Phenolic –OH
138.2	C4	Phenolic –OH
120.5	C1	Phenolic carbons
109.0	C2, C6	Phenolic carbons
**Tannic acid**		
169.7	C7	Carbonyl C=O
165.4	C7′	Carbonyl C=O
144.9	C3, C5, C3′, C5′	Phenolic –OH
139.2	C4	Phenolic –OH
138.0	C4′	Phenolic –OH
121.3	C1, C1′	Phenolic carbons
109.1	C2, C6, C2′, C6′	Phenolic carbons
92.5	C1″	Closed glucoside
71.9	C2″	Closed glucoside
67.1	C4″	Closed glucoside
66.2	C6″	Closed glucoside
63.0	C5″	Closed glucoside

^1^ Spectra were applied in Figure S1, the numbering of carbons was referred to Figure 2.

**Table 2:** Tyrosinase inhibition ability of the valonea tannin, tannic acid and gallic acid.

Sample	Valonea Tannin	Tannic Acid	Gallic Acid	Hydroquinone
IC_50_ (mM) ^1^	1.15 ± 0.37	4.00 ± 0.10	389.56 ± 4.77	1809.38 ± 5.74
IC_50_ (g/L)	3.50 ± 0.11	6.80 ± 0.17	66.23 ± 0.81	199.21 ± 0.63
Fold ^2^	--	3.4	339	

^1^ IC_50_ was the half maximal inhibitory concentration, data were expressed as mean of 3 replicates ± standard deviation; Kruskal–Wallis test showed *p* < 0.05, indicated significant differences between IC_50_ obtained from different samples. ^2^ The fold was calculated through: Fold = IC_50_ of tannic acid or gallic acid/IC_50_ of valonea tannin.

**Table 3:** Effects of Valonea tannin on tyrosinase activities ^1^.

	Valonea Tannin	Tannic Acid	Gallic Acid
Inhibition mechanism	Reversible	Reversible	Reversible
Inhibition type	Competitive-noncompetitive mixed	Competitive	Competitive
Inhibition constants (mM) ^2^	K_IS_ = 1.68 ± 0.18	K_I_ = 1.97 ± 0.32	K_I_ = 56.58 ± 3.65
	K_I_ = 0.51 ± 0.05		

^1^. Data are expressed as mean of 3 replicates ± standard deviation, the model fitting results, including R^2^ and *p* values, are shown in Table S4; Kruskal–Wallis test showed *p* < 0.05, indicated significant differences between K_I_ and K_IS_ obtained from different samples. ^2^. K_I_ was the inhibition constant, K_IS_ was the inhibition constant in enzyme−substrate complex, K_IS_ was attribute to the valonea tannin–tyrosianse-L-DOPA complex.

**Table 4:** Information of valonea tannin–tyrosinase binding process obtained by fluorescence spectra ^1^.

	Valonea Tannin	Tannic Acid	Gallic Acid
Maximum emitting wavelength (nm)	340	340	340
Quenching efficiency (%) ^2^	74.3 ± 0.2	66.5 ± 0.4	24.2 ± 0.4
K_q_ (×10^13^)	2.85 ± 0.00	1.94 ± 0.00	0.28 ± 0.00
Quenching type	Static	Static	Static
Linkage type	Non-covalent	Non-covalent	Non-covalent
K_a_ (L/mol) ×10^5^	2.23 ± 0.00	1.32 ± 0.00	0.73 ± 0.00

^1^ Data are expressed as mean of 3 replicates ± standard deviation, the model fitting results, including R^2^ and *p* values, are shown in Table S4; K_q_ and K_a_ was Stern–Volmer quenching rate constant and apparent binding constant, respectively; Kruskal–Wallis test showed *p* < 0.05 for quenching efficiency, K_q_ and K_a,_ respectively, indicated significant differences between different samples. ^2^ Calculated as: Quenching efficiency = 100 × emission at 340 nm (with 10 μM inhibitor)/emission at 340 nm (without inhibitor).

**Table 5:** Information of valonea tannin–tyrosinase binding process obtained by isothermal titration calorimetry ^1^.

	Valonea Tannin	Tannic Acid	Gallic Acid
ΔH (×10^4^ cal/mol)	−1.26 ± 0.44 (site 1)	−33.62 ± 1.11	−6.34 ± 0.23
	−6.66 ± 0.22 (site 2)		
TΔS (×10^4^ cal/mol/deg)	−0.33 (site 1)	−32.79	−5.67
	−5.90 (site 2)		
K (×10^5^)	70.6 ± 3.0 (site 1)	2.0 ± 0.5	0.9 ± 0.3
	4.3 ± 0.8 (site 2)		
n	1.02 ± 0.04 (site 1)	2.50 ± 0.05	8.75 ± 0.21
	2.70 ± 0.05 (site 2)		

^1^ Standard deviations were based on the accuracy of the curve fit to the data and obtained by Origin software package, estimated thermodynamic binding parameters for the interaction of tyrosinase and inhibitors at 298 K, ΔH and TΔS was the change in enthalpy and change in entropy, respectively, while n was the stoichiometry and K was the binding constant.

**Table 6:** Copper ion chelating and antioxidant abilities provided by valonea tannin, tannic acid and gallic acid ^1^.

	Cu^2+^ Chelating (%)	DPPH^·^ (IC_50_ mM)	ABTS^·+^ (IC_50_ mM)
Valonea tannin	56.61 ± 0.66	0.051 ± 0.002	0.040 ± 0.000
Tannic acid	56.24 ± 0.10	0.043 ± 0.001	0.043 ± 0.002
Gallic acid	52.17 ± 0.53	0.322 ± 0.004	0.253 ± 0.001
Ascorbic acid	-	0.683 ± 0.009	0.713 ± 0.012

^1^ Data are expressed as mean of 3 replicates ± standard deviation; Kruskal–Wallis test showed *p* < 0.05 for Cu^2+^ Chelating, DPPH^·^ and ABTS^·+^_,_ respectively, indicated significant differences between different samples.

## Data Availability

The data presented in this study are available on reasonable request from the corresponding author.

## Supplementary Materials

The following are available online, Figure S1. C NMR spectrum of valonea tannin, tannic acid and gallic acid; Figure S2. The tyrosinase inhibition activity evaluated by conducting the catalysis reaction with different inhibitor concentrations. Figure S3. Stern–Volmer plot (A, F_0_/F against [Q]) and double log Stern–Volmer plot (B, log [(F_0_ − F)/F] versus log [Q]) obtained from calculating valonea tannin, tannic acid and gallic acid induced fluorescence quenching of the tyrosinase. Table S4. The model fitting results from liner regression of the kinetic and fluorescence quenching analysis. Figure S5. The MALDI TOF MS spectra of the purified valonea tannin. Figure S6. The standard curve for methyl cellulose precipitation assay.

## Sample Availability

Samples of the compounds are not available from the authors.
