# Supplementary material for: Valonea Tannin: Tyrosinase Inhibition Activity, Structural Elucidation and Insights into the Inhibition Mechanism
Source: Molecules. 2021 May 7;26(9):2747. doi: 10.3390/molecules26092747 (PMC8125085; doi:10.3390/molecules26092747)
Supplement: Supplementary file 1 [file molecules-26-02747-s001.zip › molecules-1202448-supplementary.pdf]

Valonea Tannin: Tyrosinase Inhibition Activity, Structural Elucidation and Insights Into the Inhibition Mechanism

Jiaman Liu [1], Yuqing Liu [1], Xiaofeng He [1], Bo Teng [1,2] and Jacqui M. McRae [3]

[1] College of Science, Shantou University, Shantou, China; 19jmliu1@stu.edu.cn (J.L.); 15yqliu2@stu.edu.cn (Y.L.); 19xfhe@stu.edu.cn (X.H.)
[2] Guangdong Provincial Key Laboratory of Marine Biotechnology, Shantou University, Shantou, China
[3] School of Chemical Engineering and Advanced Materials, The University of Adelaide, Adelaide, Australia; jacqui.mcrae@adelaide.edu.au

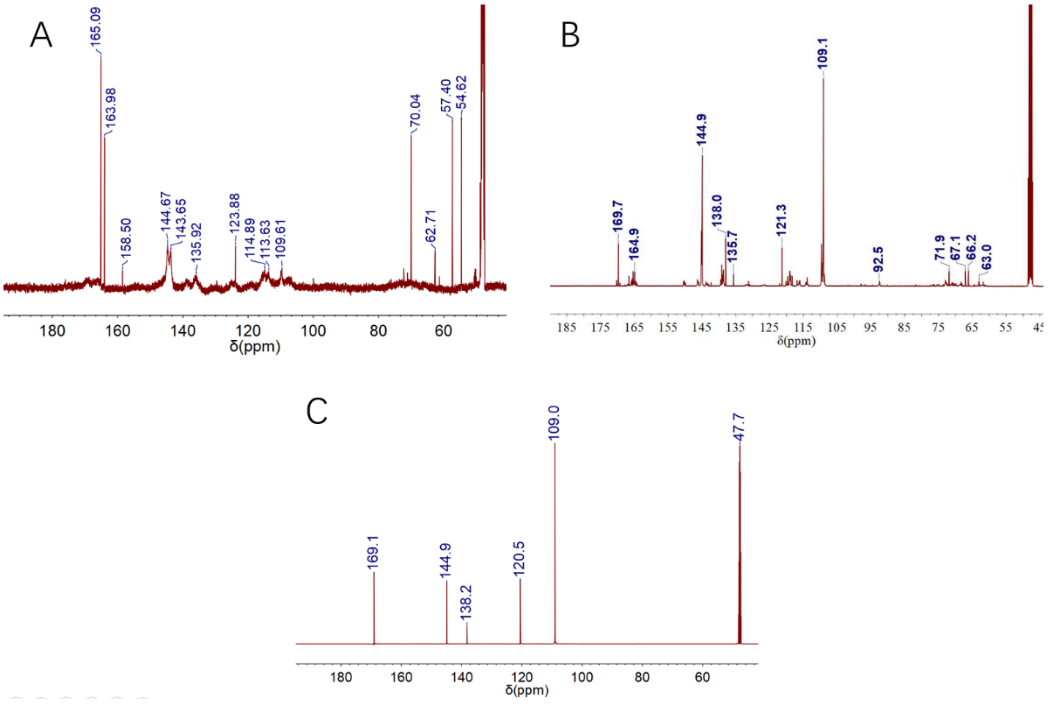

Figure S1. ^{13}CNMR spectrum of valonia tannin (**A**), tannic acid (**B**) and gallic acid (**C**).

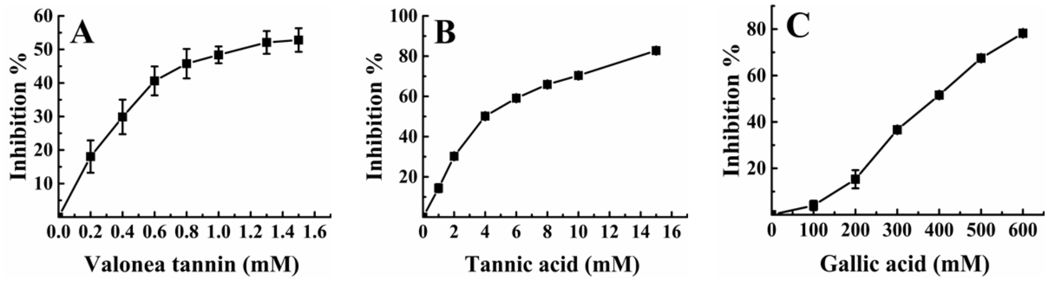

Figure S2. The tyrosinase inhibition activity was evaluated by conducting the catalysis reaction with different inhibitor concentrations. The inhibition rate-inhibitor concentration plots implied that, the inhibition rates (%) were positively related with the valonea tannin concentration (**A**), the tannic acid concentration (**B**) and the gallic acid (**C**) concentration respectively. These results implied that, the valonea tannin, tannic acid and gallic acid all presented tyrosinase inhibition ability, but their inhibitory concentration 50 (IC_{50}) were significantly different.

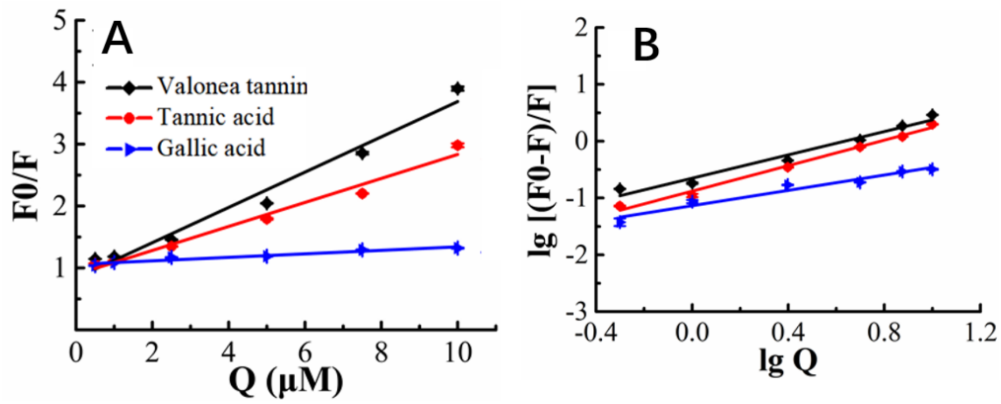

Figure S3. The Stern–Volmer plot (**A**, F0/F against [Q]) and double log Stern–Volmer plot (**B**, log[(F0 − F)/F] versus log[Q]) obtained from calculating valonia tannin, tannic acid and gallic acid induced fluorescence quenching of the tyrosinase.

Table S4. The model fitting results from liner regression of the kinetic and fluorescence quenching analysis.

**Tyrosinase concentration-reaction rate plots on Figure 4A, 4B and 4C**								
**Valonea tannin**			**Tannic acid**			**Gallic acid**		
**Line number**	**R^2**	***p***	**Line number**	**R^2**	***p***	**Line number**	**R^2**	***p***
1	99.74	<0.00001	1	99.74	<0.00001	1	99.74	<0.00001
2	99.83	<0.00001	2	99.61	<0.00001	2	99.77	<0.00001
3	99.90	<0.00001	3	99.78	<0.00001	3	99.74	<0.00001
4	99.52	<0.00003	4	99.51	<0.00001	4	99.77	<0.00001
5	99.76	<0.00001	5	99.20	<0.00003	5	99.24	<0.00003
**Lineweaver-Burk plots on Figure 4D, 4E and 4F**								
**Valonea tannin**			**Tannic acid**			**Gallic acid**		
**Line number**	**R^2**	***p***	**Line number**	**R^2**	***p***	**Line number**	**R^2**	***p***
1	98.22	<0.0007	1	95.57	<0.003	1	92.00	<0.007
2	97.20	<0.002	2	97.15	<0.001	2	96.82	<0.002
3	99.27	<0.0001	3	99.08	<0.0003	3	95.94	<0.003
4	98.49	<0.0006	4	96.05	<0.002	4	92.94	<0.006
5	94.07	<0.004	5	99.12	<0.0002	5	94.89	<0.004
**Enzyme−substrate complex (K_I) plots on Figure 4G, 4H and 4I**								
**Inhibitor**	**R^2**	***p***	**Inhibitor**	**R^2**	***p***	**Inhibitor**	**R^2**	***p***
**Valonea tannin**	93.64	<0.005	**Tannic acid**	87.98	<0.01	**Gallic acid**	92.00	<0.006
**Fluorescence quenching fitted by Stern-Volmer equation on Figure 5D**								
**Inhibitor**	**R^2**	***p***	**Inhibitor**	**R^2**	***p***	**Inhibitor**	**R^2**	***p***
**Valonea tannin**	96.95	<0.0002	**Tannic acid**	98.80	<0.00004	**Gallic acid**	94.56	<0.0007
**Stern-volmer equation fitted double logarithmic plot on Figure 5E**								
**Inhibitor**	**R^2**	***p***	**Inhibitor**	**R^2**	***p***	**Inhibitor**	**R^2**	***p***
**Valonea tannin**	97.23	<0.0003	**Tannic acid**	98.80	<0.00004	**Gallic acid**	94.56	<0.0007

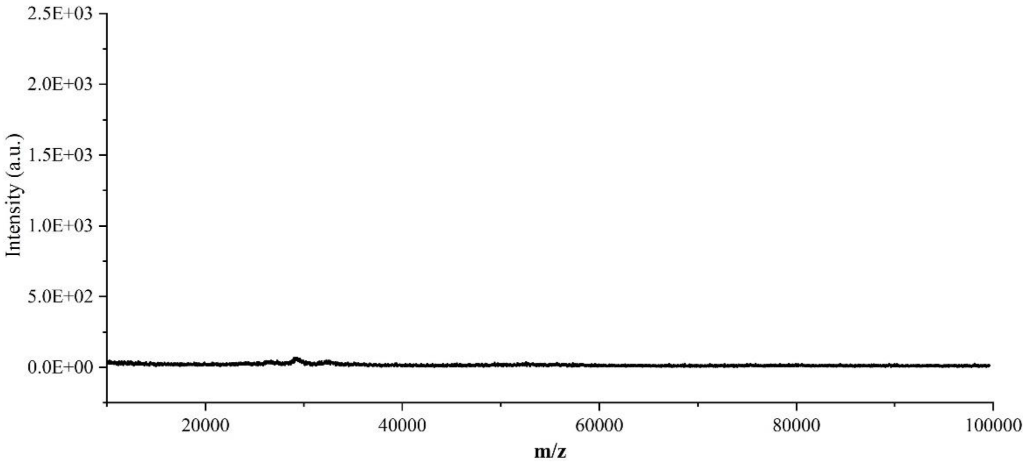

Figure S5. MALDI-TOF MS spectra of the purified tannin.

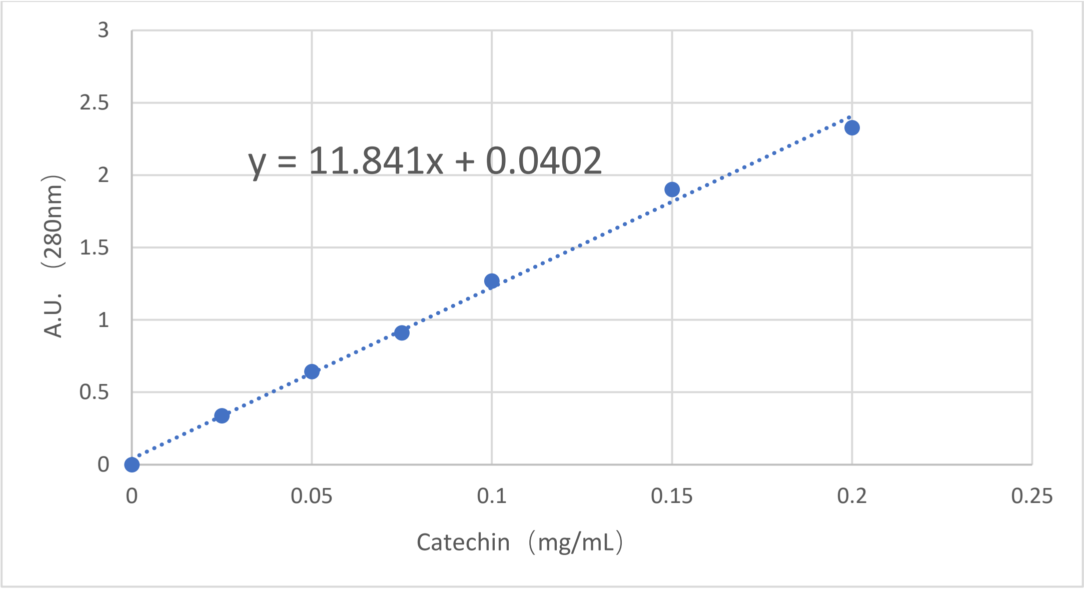

Figure S6. Standard curve of the methyl cellulose precipitation assay, where y is the absorbance at 280 nm and x is the tannin concentration (catechin equivalent).
